# Quantifying sitting posture: A pilot feasibility study of computer vision and wearable sensors (Posture Lab) using a manikin model

**DOI:** 10.1017/wtc.2025.10005

**Published:** 2025-06-16

**Authors:** Supachai Vorapojpisut, Suphawit Sansuk, Phoomtai Yindee, Darawadee Panich, Vinitha Puengtanom, Sairag Saadprai

**Affiliations:** 1 Faculty of Engineering, Thammasat School of Engineering, Thammasat University, Pathumthani, Thailand; 2 Faculty of Allied Health Sciences, Thammasat University, Pathumthani, Thailand

**Keywords:** musculoskeletal disorders, primary health care, computer vision, ArUco markers, wearable sensors

## Abstract

Posture-related musculoskeletal issues among office workers are a significant health concern, mainly due to long periods spent in static positions. This research presents a Posture Lab which is a workplace-based solution through an easy-to-use posture monitoring system, allowing employees to assess their posture. The Posture Lab focuses on two key aspects: Normal Head Posture (NHP) versus Forward Head Posture (FHP) measurement and thoracic spine kyphosis. Craniovertebral (CA) and Shoulder Angles (SA) quantify NHP and FHP. The Kyphosis Angle (KA) is for measuring normal thoracic spine and kyphosis. To measure these angles, the system uses computer vision technology with ArUco markers detection via a webcam to analyze head positions. Additionally, wearable accelerometer sensors measure kyphosis by checking the angles of inclination. The framework includes a web-based user interface for registration and specialized desktop applications for different measurement protocols. A RESTful API enables system communication and centralized data storage for reporting. The Posture Lab serves as an effective tool for organizations to evaluate employee postures and supports early intervention strategies, allowing timely referrals to healthcare providers if any potential musculoskeletal issues are identified. The Posture Lab has also shown medium to very high correlations with standard 2D motion analysis methods – Kinovea – for CA, SA, and KA in FHP with kyphosis measurements (*r* = 0.607, 0.704, and 0.992) and shown high to very high correlations in NHP with normal thoracic spine measurements (*r* = 0.809, 0.748, and 0.778), with significance at *p* < .01, utilizing the Pearson correlation coefficient.

## 1. Posture monitoring

The alignment and dynamics of human posture play a fundamental role in maintaining musculoskeletal health. Prolonged suboptimal postures and movement patterns increase the risk of musculoskeletal disorders (MSD). MSDs develop from repeated mechanical stresses – including strain, compression, and rotational forces – on muscles and skeletal structures. Such issues are particularly prevalent in workplace environments, where employees maintain static positions for prolonged durations (Singh and Singh, 2019). Environmental factors, such as workplace ergonomics and furniture configuration, can significantly contribute to postural deterioration. Beyond the workplace context, specific populations like wheelchair users face unique challenges, with research indicating a high prevalence of spinal complications (Liampas et al., 2021) due to sustained seated positions and mobility device design constraints.

**Figure 1.**
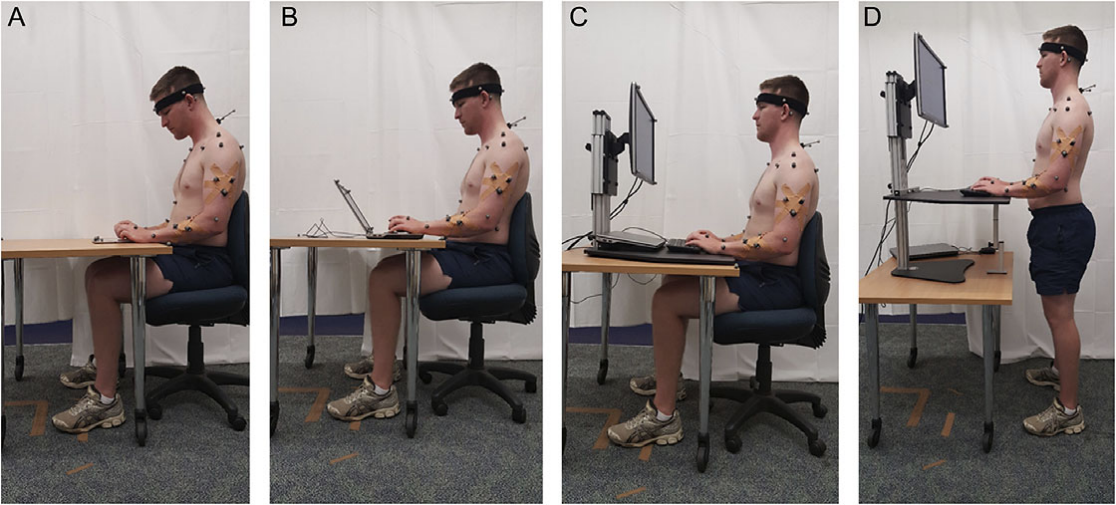
Posture monitoring experiment (reproduced from Yu et al., 2018).

The increasing digitalization of work environments and their impact on postural health forms the cornerstone of this investigation, with particular attention to seated positions commonly adopted during professional activities. The modern workplace has witnessed a surge in office syndrome manifestations, primarily attributed to extended computer-based tasks. This trend is further exacerbated by the ubiquitous use of mobile devices, leading to a phenomenon known as “tech neck” – a condition characterized by chronic neck strain from prolonged downward head positioning. Given these emerging health challenges, the systematic monitoring and evaluation of spinal alignment, particularly in the cervical and thoracic regions, has become essential for preventing musculoskeletal complications associated with contemporary lifestyle patterns. As illustrated in Figure 1, a notable study was conducted by Yu et al. (2018) to evaluate postural variations across different computing device configurations. Their research revealed that portable devices, specifically notebooks and tablets, induced more significant postural deviations – including increased neck flexion, bilateral shoulder elevation, and upper trunk flexion – compared to traditional desktop setups.

Healthcare practitioners in ergonomics, movement science, and rehabilitation rely heavily on quantifiable musculoskeletal metrics, particularly those derived from joint range-of-motion measurements. Traditional assessment methods include manual goniometry (Hancock et al., 2018), which requires direct physical contact for angle measurement, and image-based analysis software such as Kinovea (Fernández-González et al., 2020). While these approaches excel in point-in-time evaluations, they prove inadequate for continuous monitoring in workplace settings. Software tracking points often drift from their correct positions over time. As a result, evaluators are required to manually adjust tracking points throughout the session, making long-term posture monitoring cumbersome and time-consuming (Salisu et al., 2023).

Modern research in continuous posture monitoring predominantly employs sophisticated 3D motion capture technology (Yu et al., 2018; Lee et al., 2021; Lee et al., 2022). This methodology typically involves strategically positioning infrared-reflective markers on anatomical landmarks, with multiple cameras capturing synchronized imagery to reconstruct three-dimensional positional changes over time. Despite the precision and comprehensive spatial data offered by 3D motion capture systems, several practical limitations restrict their widespread adoption. These systems demand substantial financial investment, require meticulous calibration procedures, necessitate controlled laboratory environments, and depend on specially trained personnel for operation and data interpretation (Obukhov et al., 2023).

In light of these challenges with existing equipment, the researchers aim to develop a posture tracking system that simplifies and enhances usability. The proposed system should efficiently track posture, consistently monitoring desired points without losing tracking from markers. Since a single camera’s viewpoint is limited, integrating sensors helps compensate for body parts that are not easily visible, ensuring more comprehensive tracking. This improvement will eliminate the need for manual adjustments by evaluators during sessions, making long-term posture monitoring more convenient. Additionally, the system should be affordable, easy to use, and uncomplicated, allowing the employees to utilize it independently while facilitating data transfer to specialists for further analysis. This research introduces Posture Lab, a prototype system that enables real-time posture monitoring through accessible technologies. The implementation combines computer vision techniques utilizing ArUco markers, fiducial markers traditionally employed in robotic navigation (García-Ruiz et al., 2023), with wearable accelerometer sensors (Greene et al., 2022; Ali et al., 2023).

The Posture Lab focuses on two key measurements: Normal head posture (NHP) versus forward head posture (FHP) and normal thoracic spine versus kyphosis measurements. The craniovertebral angle (CA) and shoulder angle (SA) are used for measuring NHP and FHP, while the kyphosis angle (KA) assesses normal thoracic spine alignment and thoracic kyphosis. The Posture Lab employs computer vision technology with ArUco marker detection via a webcam to analyze head positions. Additionally, wearable accelerometer sensors measure kyphosis by checking the angle of inclination. The framework includes a web-based user interface for registration and specialized desktop applications for various measurement protocols. All components of the system communicate through a RESTful API, with data stored centrally for comprehensive reporting. Employees with basic musculoskeletal assessment knowledge can operate the Posture Lab. While acknowledging potential trade-offs in measurement precision, the Posture Lab prioritizes accessibility and practical implementation as a primary healthcare screening tool aimed at facilitating early detection of potential musculoskeletal complications.

Furthermore, in this study, we compared Posture Lab with Kinovea, as both systems employ single-camera 2D analysis. The aim was to evaluate the feasibility of using Posture Lab – a single camera system with markers and accelerometer sensors – to monitor human sitting postures. While Kinovea’s reliance on manual intervention limits its practicality for extended-duration assessments, it remains a convenient, valid and reliable field-testing device due to its single-camera setup and high accuracy compared to 3D motion analysis (Shishov et al., 2021; Puig-Diví et al., 2019). If the data from Posture Lab align closely with Kinovea’s results, it would suggest that Posture Lab could serve as a viable alternative for postures analysis, particularly in scenarios where single camera 2D systems like Kinovea are traditionally used.

## 2. Posture measurement techniques

The assessment of posture-related musculoskeletal disorders involves examining multiple anatomical deviations, particularly in the cervical, shoulder, and spinal regions. These physical alterations are routinely evaluated in clinical settings by physical therapy professionals. FHP and kyphosis angle have emerged as particularly valuable indicators, especially for office workers who frequently lean forward toward computer screens and wheelchair users who maintain prolonged seated positions. These metrics are primarily selected due to their quantifiable nature using conventional assessment techniques. However, existing measurement approaches present challenges for continuous monitoring needed to evaluate behavioral patterns or environmental influences in both workplace settings and daily wheelchair use. Based on these considerations, our prototype system specifically targets these two key postural parameters, which are critical indicators for both population groups.

Under optimal conditions, the head maintains vertical alignment with the spine, characterized by ear–shoulder vertical correspondence. FHP (Chu, 2022) describes a postural deviation where the cervical spine alignment is compromised, resulting in anterior head displacement. This condition frequently develops from sustained forward head tilt during computer or mobile device usage, potentially leading to muscular and skeletal stress in the cervical and upper thoracic regions. Maintaining FHP for extended periods can cause pain, stiffness, and headaches. Clinical assessment of FHP traditionally relies on observing the ear–shoulder–hip vertical alignment. Optimal posture is characterized by these anatomical landmarks forming a vertical reference line, with deviations from this alignment suggesting potential FHP development. According to contemporary research works, the CA typically measures less than 48° (Kim et al., 2018), signaling postural dysfunction and SA often exceeds 54° (Nam et al., 2013), reflecting compensatory muscular adaptations. This postural syndrome can lead to increased cervical spine strain, muscular imbalances, and potential long-term musculoskeletal complications.

**Figure 2.**
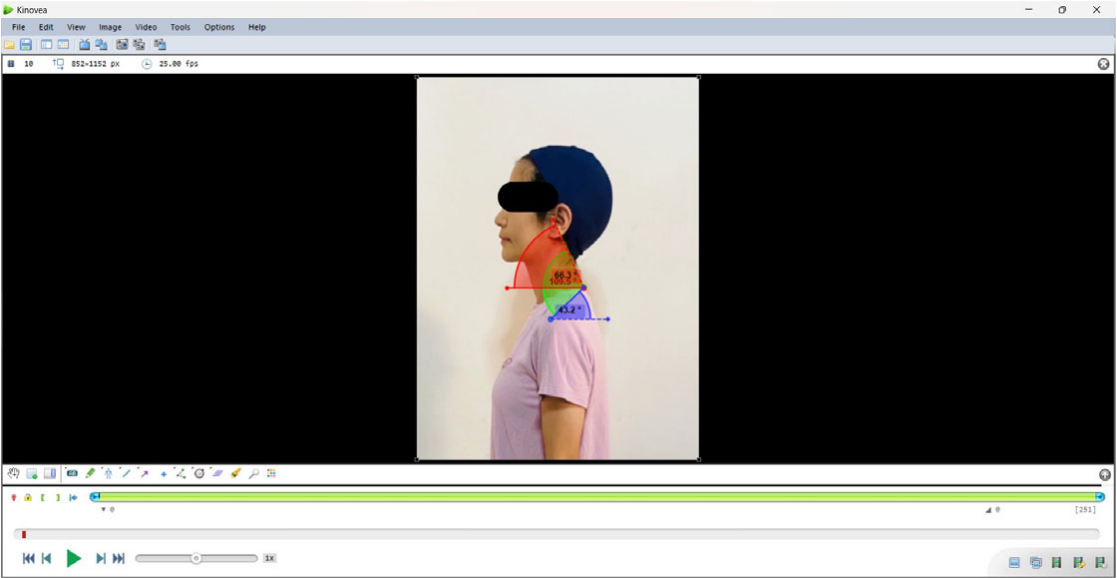
Measurement of forward head angle using Kinovea.

Our assessment methodology quantifies FHP severity through the forward head angle measurement, defined by the angular relationship among three anatomical landmarks: the tragus (ear), C7 vertebral spinous process, and acromion process of the scapula, while conventional assessment methods employ goniometry or image analysis software like Kinovea as shown in Figure 2. Although Aliaa (2016) concluded in their study that Kinovea demonstrated Kinovea’s practicality for field studies and demonstrated validated Kinovea’s utility for large-scale postural studies. Moreover, Sharifnezhad et al. (2021) concluded in their study that Kinovea demonstrates excellent interrater and intrarater repeatability for measuring kyphosis and lumbar lordosis. However, the automatic movement tracking of the Kinovea program often encounters issues with tracking the desired anatomical landmarks. It is frequently observed that the tracking deviates from the correct points, necessitating manual tracking to correct these errors. Additionally, when tracking postures over extended periods, such as analyzing sitting posture during work, there tends to be a need for manual data analysis. This can be time-consuming due to the large amount of data (Salisu et al., 2023). However, while Kinovea’s reliance on manual intervention limits its practicality, especially for extended-duration assessments, it remains a convenient, valid, and reliable field-testing device due to its single-camera setup and high accuracy compared to 3D motion analysis, which is considered the gold standard (Shishov et al., 2021; Puig-Diví et al., 2019).

**Figure 3.**
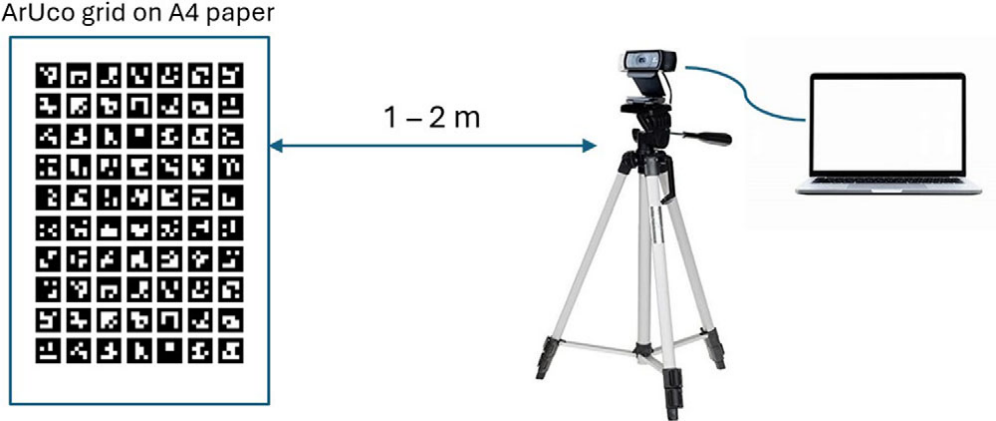
Experiments for evaluating ArUco markers.

To address the challenge of identifying subcutaneous landmarks, our approach employs a practical solution where individuals with basic training in anatomical landmarks can physically locate these points through palpation, then mark them with fiducial tags. We selected ArUco markers as the visual reference system due to their superior detection reliability compared to AprilTag, another popular fiducial tag (Zakiev et al., 2020). These markers serve as clear visual anchors that can be consistently tracked using computer vision techniques, bridging the gap between manual anatomical identification and automated measurement. The markers, once placed on identified landmarks, enable continuous tracking of postural changes through standard webcam technology. Based on consultation with physical therapists, we selected a marker size of 1 square centimeter – large enough for webcam detection while minimizing coverage of anatomical landmarks. Through experimental testing of various ArUco dictionaries – predefined sets of square markers with different bit patterns – printed as the grid on standard A4 papers, we determined as shown in Figure 3 that the 4 × 4–50 dictionary provided optimal detection at typical experimental distances (1–2 m).

Detecting ArUco markers from images captured by webcams is achieved through Python code that utilizes the OpenCV library. The ArUco API provides the list of detected ID and (*x*, *y*) coordinates of all four corners for each marker. These corner coordinates can be used to calculate the center of the marker, as shown in (1) and (2). The system then processes these marker positions to compute the forward head angle, providing quantitative assessment of FHP severity.(1)

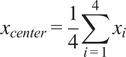


(2)

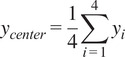



Let ( 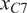 , 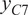 ) and ( 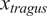 , 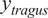 ) represent the 2D coordinates of the C7 spinous process and ear tragus markers, respectively. The horizontal vector 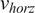 is defined as (1,0), aligned with the image’s x-axis. The vector from C7 to the tragus is 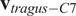 = ( 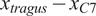 , 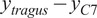 ). Then, the CA angle is computed as:(3)

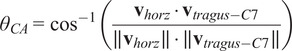



The SA is derived similarly using shoulder marker coordinates. Let ( 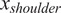 , 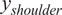 ) denote the 2D coordinates of the acromion markers. The SA is computed as:(4)

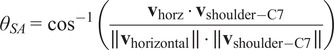



**Figure 4.**
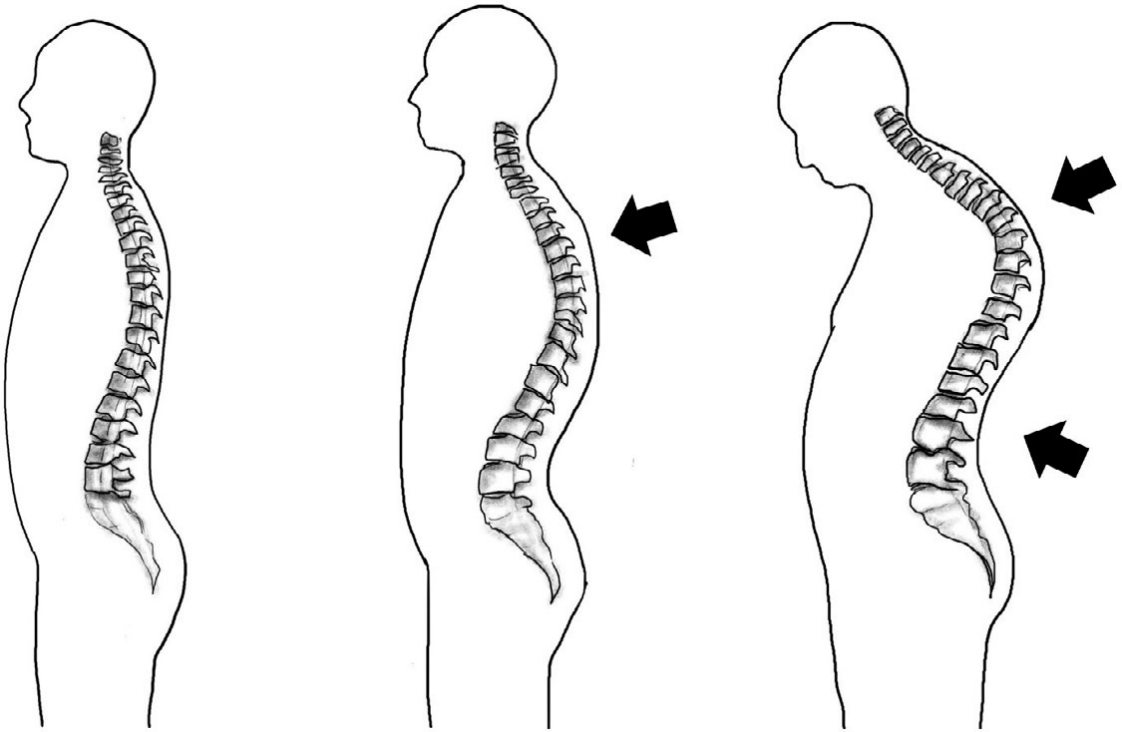
Musculoskeletal disorder (MSD) associated with kyphosis.

Kyphosis, characterized by an excessive outward curvature of the upper thoracic spine (between the neck and ribs), is another posture-related disorder often caused by prolonged poor posture, such as slouching or hunching over for extended periods. If left unattended, kyphosis can lead to deformities in the upper back, resulting in a curved or “humpback” appearance as shown in Figure 4.

The curvature of the upper back (thoracic spine) can be assessed using various indicators, such as the kyphosis angle and alpha/beta angles, with the kyphosis angle being the most commonly used technique. Traditionally, the evaluation of kyphosis has often relied on X-ray images of the spine. However, this approach is not suitable for continuous monitoring of back curvature during routine activities or in workplace environments. Therefore, there is a need for alternative methods that can accurately assess kyphosis without the limitations of X-ray imaging.

**Figure 5.**
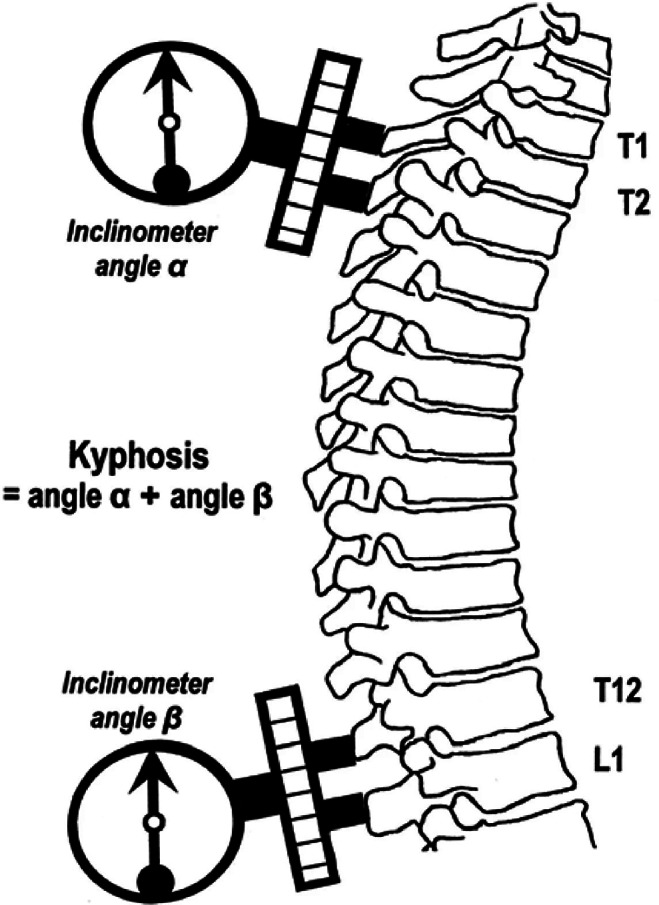
Measuring alpha and beta angles with accelerometer sensors to analyze kyphosis (reproduced from Huang et al., 2022).

The Posture Lab chose to measure the kyphosis angles (KAs) formed by the prominences at T1 and T2 (alpha angle) and at T12 and L1 (beta angle), and then add them together to obtain the desired kyphosis angle as shown in Figure 5. Although the calculation of these two angles does not directly reflect the curvature of the back, a study (Lewis and Valentine, 2010) found that the alpha, beta angles are correlated to the kyphosis angle. Our survey revealed that accelerometer sensors have been employed as digital inclinometers for measuring the alpha/beta angle, which is then presented as the kyphosis angle in both smartphone applications (Huang et al., 2022) and products such as EasyAngle. Therefore, the Posture Lab chose to use accelerometer sensors as wearable devices, allowing for continuous measurement and monitoring of the alpha and beta angles corresponding to the relevant bone landmarks. For kyphosis assessment in this study, the Posture Lab employs two wireless inclinometers which wirelessly connect to a computer via Bluetooth 5.0 protocol. The accelerometer sensors are a compact device featuring an MPU9250 as a 9-axis inertial measurement unit (IMU) with a built-in battery. The tilt angles relative to the Earth’s axis are calculated from the acceleration data along the X-axis, sampled at a rate of 1 Hz. The inclination accuracy is specified as 0.2°.

The approaches of ArUco markers and wearable devices enable the assessor to determine how the employees’ posture and behavior change over time while sitting or standing to perform assigned tasks. The continuous monitoring capability provided by wearable accelerometer sensors offers a significant advantage over traditional methods that rely on static measurements or snapshots.

## 3. System architecture

This research presents a prototype system – Posture Lab – for workplace musculoskeletal disorder (MSD) assessment, designed specifically as an accessible primary healthcare service within organizational settings. The system architecture prioritizes privacy protection, user-friendly operation, and cost-effective implementation while serving three key stakeholder groups:

- Organizations: Enables proactive workplace health management through systematic posture monitoring, supporting occupational health initiatives without significant infrastructure investment.
- Assessment Personnel: Accommodates staff with basic training in musculoskeletal assessment protocols, focusing on screening and data collection rather than clinical diagnosis, thereby reducing operational costs and training requirements.
- Employees: Provides nonintrusive posture evaluation during regular work activities, ensuring minimal disruption to daily tasks while maintaining data privacy.

Given the need to handle multiple simultaneous assessments while protecting personal information, the system implements a strategic separation between confidential registration data and posture evaluation processes.

**Figure 6.**
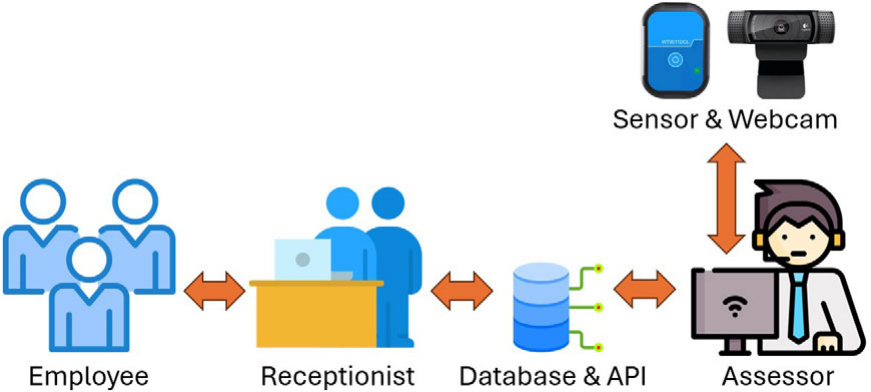
Flow of system interactions.

The flow of system interactions, illustrated in Figure 6, comprises two primary components designed for efficient healthcare service delivery. The registration component operates through a web-based platform, providing secure handling of personal information and assessment scheduling. The evaluation component runs locally on assessment station computers connected to accelerometer sensors, ensuring data privacy and reducing network security risks. These components communicate through a secure RESTful API, with all data centrally stored to facilitate comprehensive reporting while maintaining data protection standards.

**Figure 7.**
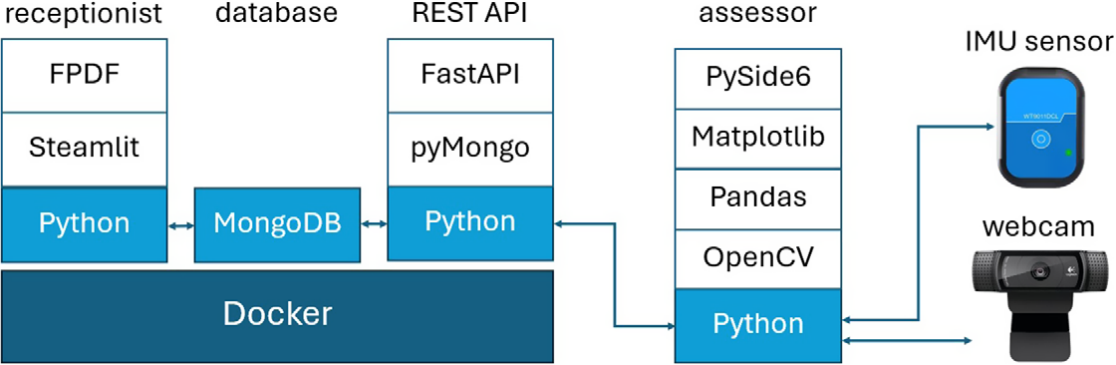
Local web-based software architecture.

As shown in Figure 7, the registration system leverages Docker containerized architecture for cost-effective deployment and maintenance:

- User interface container utilizing Python with Streamlit and FPDF libraries, ensuring intuitive operation for nontechnical healthcare staff.
- API container employing FastAPI for secure, efficient data exchange.
- MongoDB container providing scalable, privacy-compliant data storage.

The database structure incorporates three distinct collections, segregating personal information, assessment requests, and evaluation results to enhance data security. The API implementation features two dedicated channels: a GET channel for assessment station job assignments and a POST channel for results submission. System deployment utilizes Docker Compose for streamlined installation and operation, minimizing IT support requirements and maintenance costs.

The assessment stations feature dedicated desktop applications developed using Python with PySide6, each tailored to specific postural measurements. The FHP assessment station utilizes webcam technology to track ArUco markers for forward head angle calculations, while the kyphosis station employs wireless accelerometers to measure gravitational inclination for alpha/beta angle determinations.

**Figure 8.**
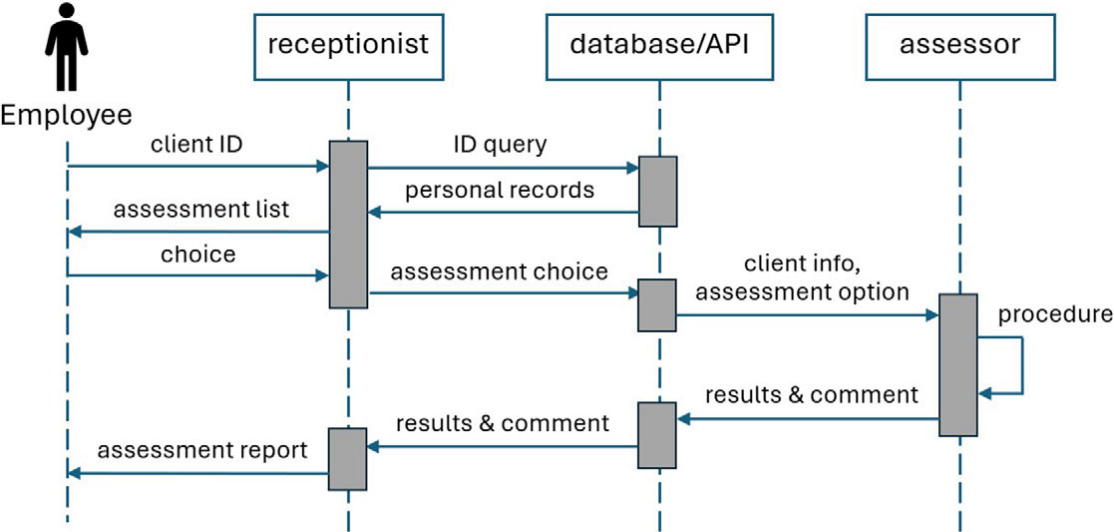
The assessment process.

Figure 8 outlines the user journey through the assessment process. Employees begin by either completing initial registration or accessing their existing profile through a previously assigned identifier. After selecting their desired assessment type, the evaluation station retrieves their information and displays it for the attending staff member. The assessment process emphasizes clear communication, with staff members providing detailed explanations of procedures before beginning measurements. During the evaluation period, participants perform specified activities while the system continuously monitors their postural metrics, with real-time trending displayed for the evaluator’s reference.

Upon completion of the assessment, assessor conducts a preliminary analysis of the collected posture data and provide initial recommendations based on observed patterns. This information is securely transmitted to the central system through the REST API. The process concludes with the generation of a comprehensive PDF report at the registration area, detailing the assessment findings.

This document serves dual purposes: providing immediate feedback to the employees while also serving as a standardized reference for healthcare professionals, such as physical therapists, movement scientists or orthopedic specialists, who may conduct more detailed clinical evaluations including advanced imaging studies.

**Figure 9.**
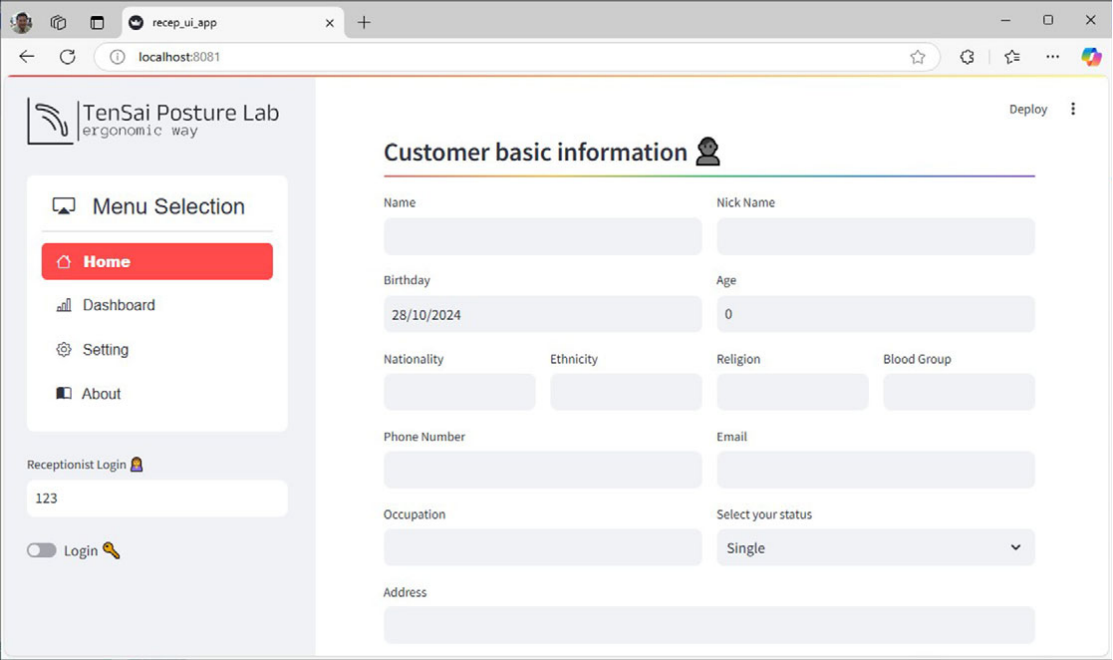
Web-based UI for registration stage.

The Web-based User Interface for Registration Stage, illustrated in Figure 9, demonstrates the system’s user-centric approach. The web-based platform guides users through a streamlined process, differentiating between first-time and returning employees. New users complete a profile including essential health metrics such as weight and height, receiving a unique identifier for future visits. Returning users can efficiently access and update their existing information before proceeding to schedule their assessment.

**Figure 10.**
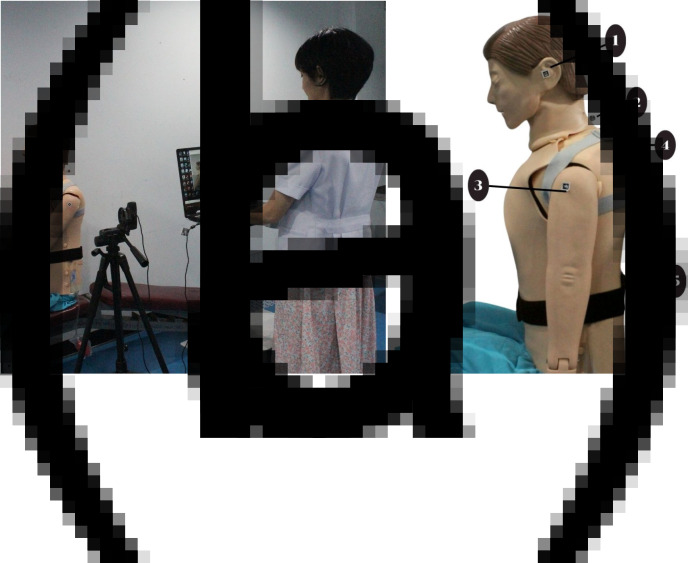
(a) FHP and kyphosis assessment station, (b) position of the markers and accelerometer sensors: (1) ear, (2) lower neck, (3) shoulder, (4) upper back, (5) mid-back positions.

**Figure 11.**
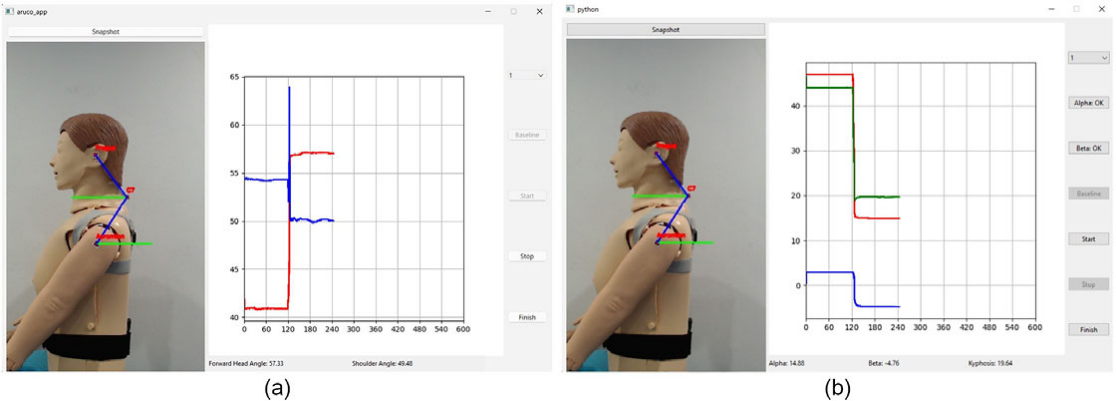
User interface of (a) FHP and (b) kyphosis assessment.

The prototype system of Posture Lab features FHP and kyphosis assessment stations as shown in Figure 10(a) The FHP assessment station, which is connected to a Logitech C920 webcam with a resolution of 2048 × 1080 pixels. The webcam is positioned in portrait orientation and placed approximately 1 m away from the person under assessment and the kyphosis assessment station using two WitMotion WT9011DCL-BT50 devices as wireless inclinometers. Two tasks (reading documents on iPad and notebook computer) were given as the computer software at the FHP and kyphosis assessment stations import the video feed and detects ArUco markers and accelerometer sensors. The positions of the ArUco markers are at numbered 1 (ear position: tragus of the ear), numbered 2 (lower neck position: spinous process of C7 vertebra), and numbered 3 (shoulder position: acromion process of the scapula). For the kyphosis assessment station, the positions of the two wireless inclinometers are at numbered 4 (upper back position: T1 and T2 vertebrae) and numbered 5 (mid-back position: T12 and L1 vertebrae) as shown in Figure 10(b). Using the ArUco markers and accelerometer sensors, the software calculates and continuously displays the forward head angle and kyphosis on the screen every second and the evaluation time can be custom-selected. The user interface of FHP assessment is displayed in Figure 11(a), which shows FHP data on a screen. On the right side of the screen, there is a graph with the x-axis representing CA (red line) and SA (blue line) in degrees and the y-axis representing time in seconds. The left side of the screen features an image where the system creates a hypothetical line (blue lines) from the markers at the ear position to the lower neck position and connects to the shoulder position. Additionally, it generates another hypothetical line (green lines), which is a horizontal plane cutting between the marker positions at the lower neck and shoulder, forming angles CA and SA. Moreover, the user interface of kyphosis assessment is displayed in Figure 11(b), which shows kyphosis data on a screen. On the right side of the screen, there is a graph with the x-axis representing alpha (red line), beta (blue line), and kyphosis (green line) angles in degrees and the y-axis representing time in seconds. The left side of the screen features an image; however, the system does not create any hypothetical line. The Posture Lab outputs an assessment report with graphs and images and raw data in Excel files for CA, SA, and KA values, allowing experts to analyze FHP and kyphosis further.

**Figure 12.**
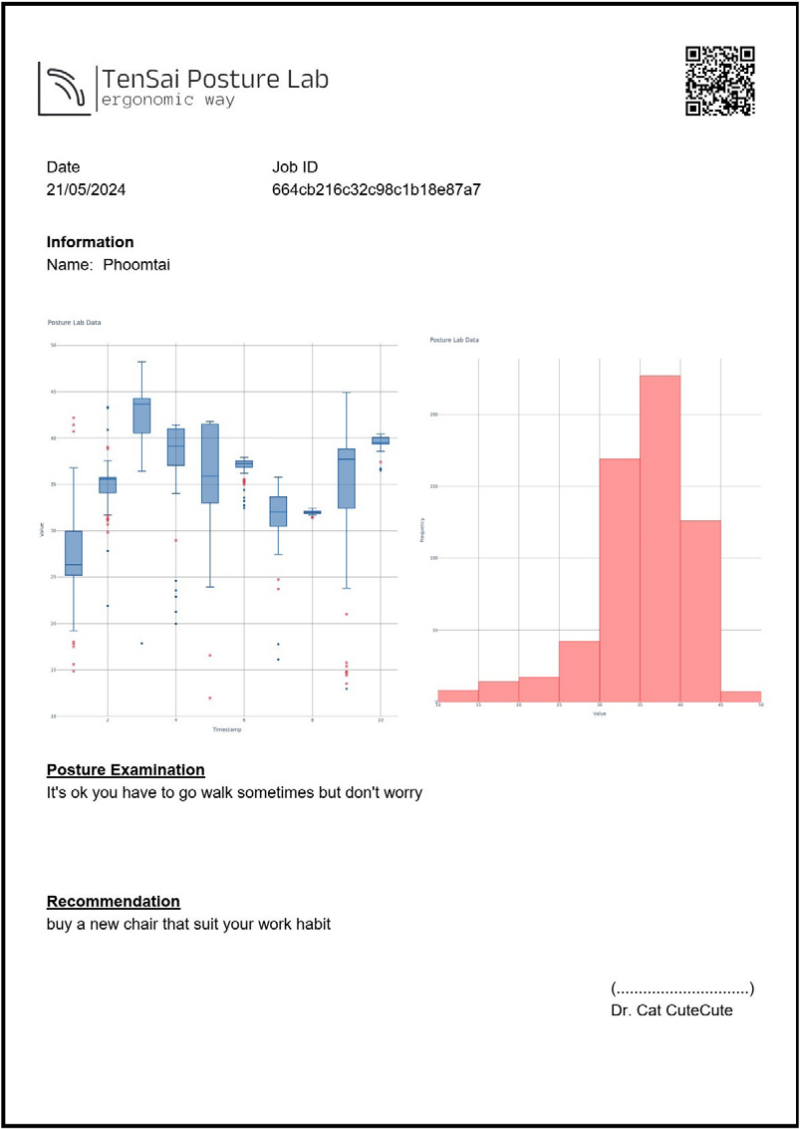
Example of assessment report.

The example of an assessment report, as shown in Figure 12, is developed using the FPDF library based on data and assessment status. The collected data are aggregated on a per-minute basis and presented in the form of a box plot, displaying the trend of indicator changes over time. Additionally, a histogram is included to illustrate the distribution of indicator values. Compared to traditional snapshot-based posture indicator measurements, the inclusion of trend and distribution information enables healthcare professionals to assess the progression of MSD and posture status in near-real-life environments. This comprehensive information allows for a more informed evaluation, leading to the development of effective intervention for behavior modification or appropriate treatment recommendations.

## 4. Material and methods

To validate the Posture Lab prototype’s effectiveness, the researchers conducted comparative accuracy assessments against established 2D standard motion analysis software – Kinovea, using standardized measurement protocols on a physical manikin model. Kinovea is a convenient, valid, and reliable field-testing device due to its single-camera setup and high accuracy compared to 3D motion analysis, which is considered the gold standard (Shishov et al., 2021; Puig-Diví et al., 2019). The reason the researchers chose to work with a manikin is that it allows for the development of a prototype innovation for measuring angles while sitting, which will be used to analyze FHP and kyphosis. The researchers aim to conduct preliminary tests to assess the feasibility and safety of this approach before applying it in real humans. In the future, the researchers plan to seek ethical approval for human research to test the practical application of this method in order to assess the feasibility of analyzing FHP and kyphosis in humans because issues related to human posture can encompass a variety of factors, such as scoliosis, pelvic tilt, and more. Therefore, further studies are necessary to gain a comprehensive understanding of these conditions.

**Figure 13.**
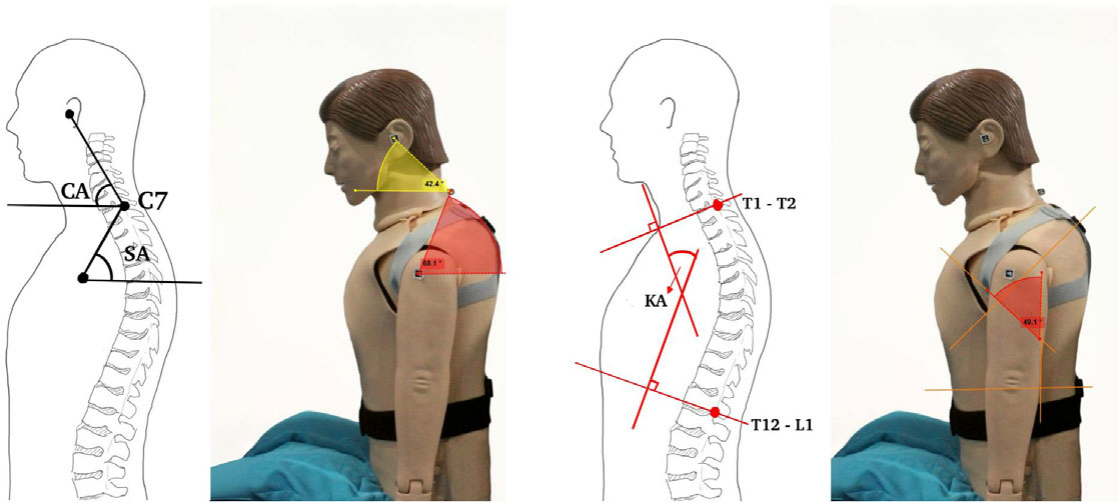
Positioning of the manikin for CA, SA, and KA analysis.

The experiment began by positioning the manikin model to sit on the seat in two postures. The manikin (DM-CPR2300) measures about 157 cm in height and 52 cm in width and approximately 24 kg in weight, with realistic body proportions and joint mobility. The first posture, referred to as FHP with kyphosis, positions the model’s head forward relative to the body’s vertical midline. In this posture, the ears are in front of this alignment, indicating a forward head position. The researchers adjusted the CA to 40°, the SA to 55°, and the KA to 45°, as measured with a goniometer (see Figure 13). In contrast, the second posture, known as NHP with normal thoracic spine, aligns the model’s ears with the shoulder and midline, ensuring that the visual axis is as horizontal as possible. The researchers adjusted the CA to 55°, the SA to 50°, and the KA to 20°. This alignment is measured using a goniometer. According to established criteria, the angles characteristic of FHP with kyphosis are defined as a CA of less than 48° (Kim et al., 2018), an SA greater than 54° (Nam et al., 2013), and an KA greater than 40° (Koelé et al., 2020).

Each posture was monitored for 120 s using Posture Lab and Kinovea which measure continuously for a total of 240 s which is a sufficient duration to observe the feasibility of monitoring sitting posture (Kappattanavar et al., 2021), sampled at a rate of 1 Hz. Measurements for postures were taken six measurements (the first measurement for practice and the second to sixth measurements for data collection). The researchers began by adjusting a manikin to sit in FHP with kyphosis position for static measurement. Then marking a dynamic measurement phase from second 120 onward, the posture was changed to NHP with normal thoracic alignment for static measurement. The researchers adjusted both the FHP with kyphosis and NHP with normal thoracic by the goniometer. Therefore, the dynamics changes in angles can be observed during approximately since seconds 120. Once the manikin remained in the NHP with normal thoracic spine position, it constituted another static measurement until data collection ended at second 240. During the measurement, the researchers measured the postures by recording a webcam (Logitech C920 webcam with a resolution of 2048 × 1080 pixels) held in portrait mode to capture the head positions on the left side of the model. The webcam is positioned in portrait orientation and placed approximately 1 m away from the manikin. To facilitate FHP analysis, ArUco markers were attached to the manikin’s anatomical landmarks in the sagittal plane using tape: at numbered 1 (ear position: tragus of the ear), numbered 2 (lower neck position: spinous process of C7 vertebra), and numbered 3 (shoulder position: acromion process of the scapula). For kyphosis analysis, the two wireless inclinometer sensors were attached to the manikin’s spine using elastic bands specifically designed for mounting these inclinometer sensors. These specially designed elastic bands can be adjusted to position the inclinometer sensors appropriately for each individual user. The inclinometer sensors were at numbered 4 (upper back position: T1 and T2 vertebrae) and numbered 5 (mid-back position: T12 and L1 vertebrae) as shown in Figure 10(b). After recording, the video files (sampled at a rate of 1 Hz) were analyzed to both Posture Lab and Kinovea for a detailed analysis of SA, CA, and KA.

The average SA, CA and KA measurements in FHP with kyphosis and NHP with normal thoracic spine from both Posture Lab and Kinovea were calculated to examine the validity of the criterion-related validity with find correlation on validity by using Pearson correlation coefficient. The correlation coefficient *r* of 0.900–1.000 referred to a very high correlation, 0.700–0.890 referred to a high correlation, 0.500–0.690 referred to a moderate correlation, and <0.500 referred to a low correlation (Portney, 2020) at the confidence interval of 95% and statistical significance was set at *p* < .01, Sig. (two-tailed). If the statistical error is 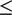 5%, this can indicate that the measurements of the SA, CA, and KA angle errors in Posture Lab were acceptable with excellent agreement when compared to those obtained from Kinovea (common in high-precision fields like biomechanics or engineering) (Hindle et al., 2020).(5)

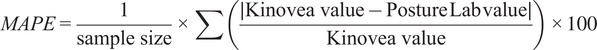



## 5. Results

The validity of the measurements taken using Posture Lab and Kinovea was evaluated through criterion-related validity, utilizing the Pearson correlation coefficient.

The results indicated that Posture Lab measurements for FHP with kyphosis posture yielded average angles for CA, SA, and KA of 41.793 degrees (SD = 1.557), 57.926 degrees (SD = 0.811), and 45.371 degrees (SD = 4.050), respectively. In contrast, Kinovea measurement for FHP with kyphosis posture reported average angles for CA, SA, and KA of 42.039 degrees (SD = 1.857), 57.956 degrees (SD = 0.894), and 45.112 degrees (SD = 4.233), respectively.

The analysis of FHP with kyphosis measurement revealed a moderate to very high correlation between Posture Lab and Kinovea. For CA, SA, and KA, the correlation coefficients were *r* = 0.607, 0.704, and 0.992, respectively, which were statistically significant at *p* < .01. The study demonstrated very good predictive performance, with MAPE values of 0.117, 0.011, and 0.115, and a corresponding coefficient of determination ( 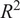 ) values of 99.80%, 64.90%, and 97.70%, respectively.

The results indicated that Posture Lab measurements for NHP with normal thoracic spine posture yielded average angles for CA, SA, and KA of 56.047 degrees (SD = 2.607), 52.779 degrees (SD = 0.404), and 20.811 degrees (SD = 0.995), respectively. In contrast, for NHP with normal thoracic spine posture, Kinovea reported average angles for CA, SA, and KA of 56.228 degrees (SD = 2.485), 52.894 degrees (SD = 0.483), and 21.230 degrees (SD = 1.625), respectively.

The analysis of NHP with normal thoracic spine measurement revealed a high to very high correlation between the Posture Lab and Kinovea. For CA, SA, and KA, the correlation coefficients were *r* = 0.809, 0.748, and 0.778, respectively, which were statistically significant at *p* < .01. The study demonstrated very good predictive performance, with MAPE values of 0.064, 0.043, and 0.395, and a corresponding coefficient of determination ( 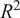 ) values of 29.90%, 56.30%, and 97.60%, respectively.

**Figure 14.**
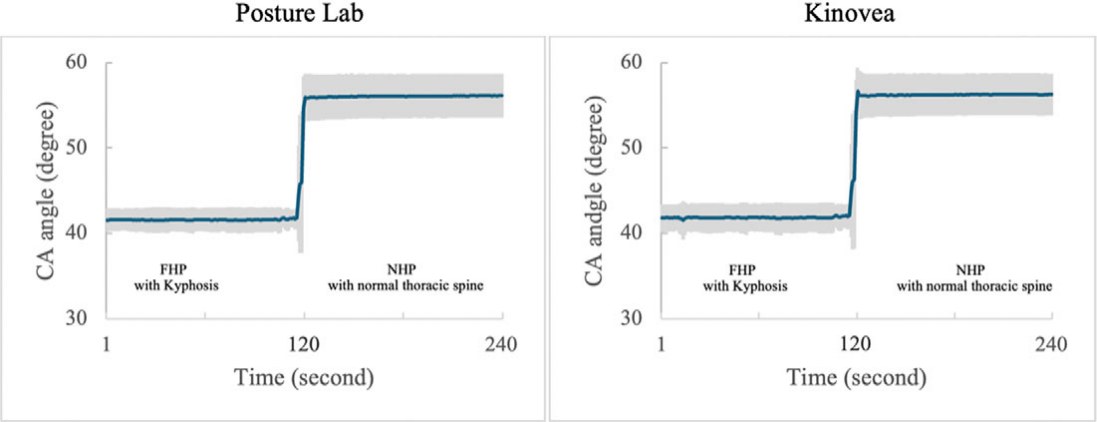
CA measurements from Posture Lab and Kinovea for FHP with kyphosis versus NHP with normal thoracic spine.

**Figure 15.**
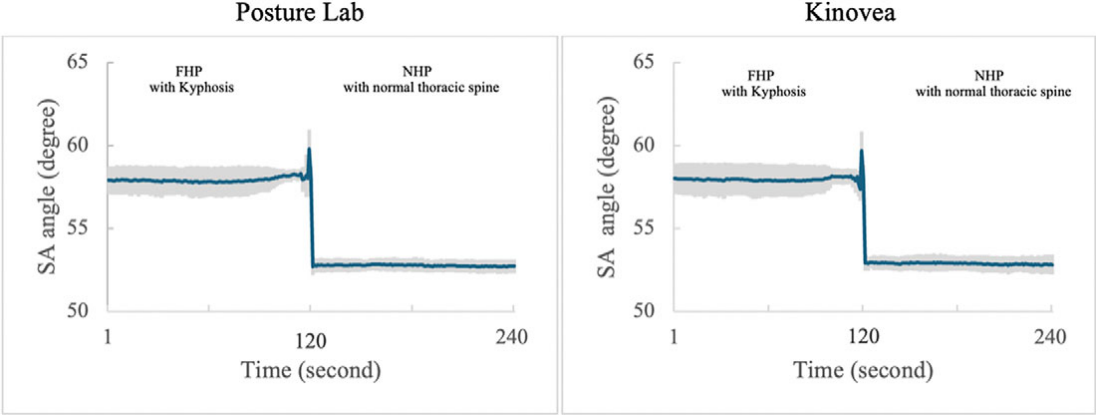
SA measurements from Posture Lab and Kinovea for FHP with kyphosis versus NHP with normal thoracic spine.

**Figure 16.**
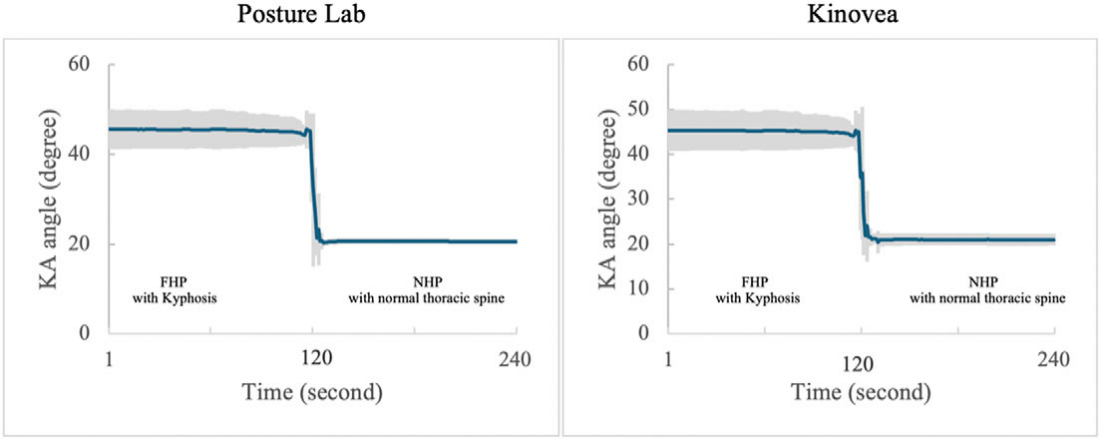
KA measurements from Posture Lab and Kinovea for FHP with kyphosis versus NHP with normal thoracic spine.

These findings demonstrate that Posture Lab provides valid measurements for assessing FHP with kyphosis and NHP with normal thoracic spine compared to Kinovea effectively as shown in Table 1. which presents the results from Posture Lab and Kinovea measurements for FHP with kyphosis and NHP with normal thoracic spine and Figures 14–16 show the graphs of CA, SA, and KA measurements from Posture Lab and Kinovea for FHP with kyphosis versus NHP with normal thoracic spine. The graphs are showing actual test results from this research. The researchers created these graphs by exporting data from the system, displaying measurements of CA, SA, and KA. The researchers began by adjusting a manikin to sit in FHP with kyphosis for static measurement. From second 120 onward, the posture was changed to NHP with normal thoracic spine, marking a dynamic measurement phase. The changes in angles can be observed since second 120 until 125. Once the manikin remained in the NHP with normal thoracic spine, it constituted another static measurement until data collection ended at second 240.

**Table 1.** Results from Posture Lab and Kinovea measurements for NHP with a normal thoracic spine and FHP with kyphosis

		FHP with kyphosis		NHP with normal thoracic spine	
Angle	Parameters	Posture Lab	Kinovea	Posture Lab	Kinovea
Craniocervical angle	Mean (SD)	41.793 (1.557)	42.039 (1.857)	56.047 (2.607)	56.228 (2.485)
	*r*	0.607*		0.809*	
	MAPE	0.117		0.064	
Shoulder angle	Mean (SD)	57.926 (0.811)	57.956 (0.894)	52.779 (0.404)	52.894 (0.483)
	*r*	0.704*		0.748*	
	MAPE	0.011		0.043	
Angle of Kyphosis	Mean (SD)	45.371 (4.050)	45.112 (4.233)	20.811 (0.995)	21.230 (1.625)
	*r*	0.992*		0.778*	
	MAPE	0.115		0.395	

* Significant at 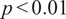 (Sig. two-tailed).

## 6. Discussion

This study found that CA, SA, and KA measured with the Posture Lab and Kinovea in FHP with kyphosis and NHP with normal thoracic spine demonstrated very good predictive performance, with MAPE values and validity at a medium to very high level for FHP with kyphosis measurement and high to very high level for NHP with normal thoracic spine. These results align with Wieczorek et al. (2020), who found ArUco markers provided accurate trajectory representation with relative errors below 5%. Similarly, Saadprai et al. (2022) demonstrated high validity, high intrarater reliability, and high interrater reliability of ArUco markers in maximum knee flexion and maximum knee extension measurement. Moreover, Godfrey et al. (2008) on Direct Measurement of Human Movement by Accelerometry found that direct measurement by accelerometry has seen the introduction of the successful implementation of low-power, low-cost electronic sensors that have been employed in clinical and home environments for the constant monitoring of patients. The qualitative and quantitative data provided by these sensors make it possible for engineers, clinicians, and physicians to work together to be able to help their patients overcome their physical disability. Furthermore, Justin et al. (2008) on Accelerometry: a Technique for Quantifying Movement Patterns during Walking found that the output of accelerometer sensors attached to the upper body has provided useful insights into the motor control of normal walking, age-related differences in dynamic postural control, and gait patterns in people with movement disorders. Previous research supports our findings on the effectiveness of ArUco markers and accelerometer sensors for motion detection. These technologies have demonstrated validity and reliability across various clinical applications, from gait analysis to postural assessment, making them suitable for clinical implementation.

In addition, Posture Lab has high to very high validity in NHP with a normal thoracic spine posture and FHP with kyphosis posture measurement because the researchers set the steps as follows. First, the researchers gave suggestions how to use the Posture Lab and Kinovea until the rater was skillful in using both devices before starting the study. Second, the rater had to practice how to correctly place the ArUco markers and accelerometer sensors at specific anatomical landmarks: the tragus of the ear, the spinous process of the C7 vertebra and the acromion process of the scapula for measuring the SA and CA and at the between of T1 and T2 vertebrae and at the between of T12 and L1 vertebrae for measuring the KA to reduce errors in posture measurement. Third, the laboratory for collecting data was spacious with enough light. Lastly, the researchers set the positions to take photos with a good resolution of the camera which is positioned in portrait orientation and placed 1 m away from the scene under assessment to show upper body while collecting the data. So, the researchers suggest that the users should follow the same or similar instructions to reduce error and produce valid and reliable data.

Additionally, this study utilized a manikin model for testing, which provided high validity since the model’s posture could be controlled precisely according to the researchers’ requirements. Nevertheless, transitioning the manikin model from FHP with kyphosis to NHP with normal thoracic spine was performed manually by the researchers. This process may have introduced unnatural movement due to hand adjustments. Consequently, this movement is not as natural as actual human posture changes. Unlike the manikin model, a real human has varied and uncontrolled conditions such as uncontrolled and continuous body movement, body shapes, and skin humidity. These conditions can affect or disturb marker attachment and measurement. Thus, using the manikin model cannot ensure the effectiveness of the proposed method. Though, the limitation of this study is conducted for preliminary tests to assess the feasibility and safety of this approach before applying it to real humans, this study is only a feasibility assessment of the device’s application and did not apply for ethical approval; therefore, testing on human subjects could not be conducted. However, in the future, the researchers plan to seek ethical approval for human research to test the practical application of this method in order to assess the analysis of FHP and Kyphosis in humans with the number of cases and include more variation of experimental settings. The issues related to human posture can encompass a variety of factors, such as muscle fatigue, proprioception, scoliosis, pelvic tilt, and more. Therefore, further studies are necessary to gain a comprehensive understanding of these conditions.

Furthermore, the ArUco markers are attached to the manikin using tape, which may not be stable enough and could easily cause the markers to fall off (the duration for each attachment is approximately 60 min). However, this issue does not affect the accuracy of the research because the researchers address it by replacing the tape each time before both the practice tests and the actual tests, ensuring that the markers do not detach during testing. The precision and consistency of markers placement are important factors of measurement accuracy. To prevent markers from falling off or shifting, this study proposes replacing the tape before each test. This approach was adopted because the researchers were concerned that the plastic material of the manikin might cause the tape to slip or detach during testing. However, in future applications involving human subjects, this issue can be mitigated by using medical tape specifically designed for 3D motion analysis such as using microporous tape which adheres securely to human skin, ensures safety, and prevents marker displacement.

Moreover, this system requires attaching the markers and accelerometer sensors to the participant’s body to measure sitting posture. If the participant wears thick clothing, or has a scarf, pillow, or other accessories, it may affect the performance of the algorithm used in this study. Therefore, the researchers recommend that future participants wear thin, form-fitting clothing without any accessories, scarves, or additional items during testing in order to obtain results that are accurate, reliable, and clear.

## 7. Conclusion

This research presents a workplace-based solution through an easy-to-use posture monitoring system, allowing employees to assess their posture. The system focuses on two measurements: NHP with normal thoracic spine measurements and FHP with kyphosis measurements, which include SA and CA for measuring NHP and FHP, and KA for measuring normal thoracic spine and thoracic kyphosis. The system uses computer vision technology with ArUco marker detection via a webcam to analyze head positions. Additionally, wearable accelerometer sensors measure kyphosis by checking the angle of inclination. The framework includes a web-based user interface for registration and desktop applications for different measurement protocols. All system components communicate through a RESTful API, and data are stored centrally for comprehensive reporting.

It demonstrated very good predictive performance, with MAPE values and the validity at the medium to very high level for FHP with kyphosis measurement and high to very high level for NHP with normal thoracic spine between Posture Lab and Kinovea. In the future, the Posture Lab can serve as a device for organizations to evaluate employee postures and supports early intervention strategies, allowing timely referrals to healthcare providers if any potential musculoskeletal issues are identified. However, this research involves a pilot test with a manikin model, but data collection from a model may not accurately reflect data collection from real humans. Future developments will focus on expanding the system’s capabilities to monitor a wider range of posture indicators in real human, allowing for more comprehensive posture assessments in various work environments among employees.
